# Supplementary material for: Electric field control of radiative heat transfer in a superconducting circuit
Source: Nat Commun. 2020 Aug 28;11:4326. doi: 10.1038/s41467-020-18163-8 (PMC7455700; doi:10.1038/s41467-020-18163-8)
Supplement: Supplementary file 1 — Supplementary Information [file 41467_2020_18163_MOESM1_ESM.pdf]

Supplementary Information for "Electric field control of radiative heat transfer in a superconducting circuit"

Maillet *et al.*
QTF Centre of Excellence, Department of Applied Physics,
Aalto University School of Science, P.O. Box 13500, 00076 Aalto, Finland

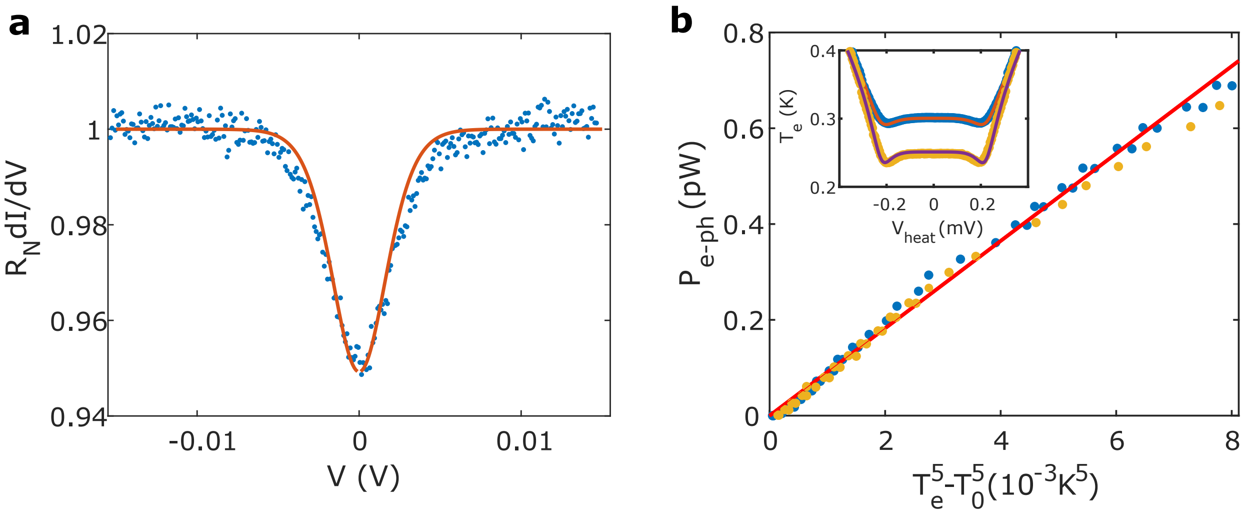

Supplementary Figure 1. **Measurement of charging energy and electron-phonon coupling constant.** a) Differential conductance of the CPT in normal state at 4.21 K, normalized to the large bias ohmic value $1/R_{\rm N}$. The red curve is the application of the universal formula derived in [1] for $E_{\rm c} = k_{\rm B} \times 0.64$ K. b) Power dissipated in the source resistor through NIS heating versus the quantity $T_{\rm e}^5 - T_0^5$, where $T_{\rm e}$ is the source electronic temperature, for cryostat temperatures $T_0 = 250$ mK (yellow dots) and 300 mK (blue dots). The fitted slope yields $\Sigma\Omega$. Inset: electron temperature in the source resistor measured with NIS thermometry versus voltage applied to another NIS junction of the source at 250 and 300 mK cryostat temperatures (same conventions). Solid lines are the application of the formula for injected power in the NIS junction (see Supplementary References [2, 3])

.

SUPPLEMENTARY METHODS

Experiment parameters. The superconducting gap, tunnel resistances of the CPT and NIS junctions were obtained through standard current-voltage characteristic measurements. The superconducting gap, assumed equal for both Aluminium layers having the same thickness and evaporated with the same target, was measured to be $\Delta = 214$ μeV $= k_{\rm B} \times 2.48$ K. The NIS probes tunnel resistances were found between 5 and 20 kΩ, while the series connection of Josephson junctions yields a normal-state resistance $R_{\rm N} = 22.5$ kΩ, from which we deduce the total Josephson energy $2E_{\rm J} = E_{\rm J1} + E_{\rm J2} = k_{\rm B} \times 1.38$ K assuming identical junctions and using the Ambegaokar-Baratoff relation for a single junction $E_{\rm J} = h\Delta/4e^2 R_{\rm N}$ (here each junction has a normal state resistance $R_{\rm N}/2$).

The single-electron charging energy $E_{\rm c} = e^2/2C$ of the CPT was determined by measuring the differential conductance of the system in weak Coulomb blockade regime in the normal state at 4.21 K. In the regime $E_{\rm c} \ll k_{\rm B}T$, the zero-bias differential conductance takes a universal value [1], $R_{\rm N} {\rm d}I/{\rm d}V|_{V=0} = 1 - E_{\rm c}/3k_{\rm B}T$, which yields a charging energy $E_{\rm c} = k_{\rm B} \times 0.64 \pm 0.05$ K [see Supplementary Figure 1a)]. Note that this is the single electron charging energy, whereas in the superconducting state the relevant energy scale is $4E_{\rm c} = (2e)^2/2C$ which is bigger than $2E_{\rm J}$. However for consistency with later steps we keep the single-electron definition for $E_{\rm c}$. By measuring both switching current (see below) and output current gate modulation for voltages $V \sim 4\Delta/e$ we obtain the $1e$ gate voltage period $\Delta V_{{\rm g},1e} = 13$ mV and deduce the gate capacitance $C_{\rm g} \approx 12$ aF.

The source and drain resistances were estimated at 4 K via four-probe measurements to be $R_i \approx 290 \pm 30$ Ω. The large uncertainty is due to the use of NIS junctions as voltage probes that measure only a fraction of the voltage drop in the resistor due to their location, as well as deviations from nominal profiles of the films and proximity superconductivity that could manifest below 1 K. The value used in the theoretical modelling of the main text is 275 Ω, well within the range expected.

The value $\Sigma = (3.7 \pm 0.2) \times 10^9$ W.K^{-5}.m^{-3} for electron-phonon coupling quoted in the main text is slightly larger than the value commonly given in the literature (2×10^9 W.K^{-5}.m^{-3}, see e.g. Supplementary References [4–6]) for thicker films but already reported previously [2] and measured independently here. As the volume Ω of the metallic piece is not known with very good accuracy, the value of Σ may not be entirely reliable, as it depends on the value chosen for Ω. We stress that this does not affect the measurements, the only experimentally relevant quantity being $\Sigma\Omega$. In Supplementary Figure 1b) we show the power dissipated in the resistors [2, 3] as a function of the electronic temperature T_e resulting from dissipation for two cryostat temperatures $T_0 = 250$ mK and 300 mK. We use such elevated bath temperatures to minimize the impact of electron-photon coupling and external heat loads.

We evaluate the parasitic external heat loads to be ≈ 2 fW, in line with similar experimental conditions [7], which

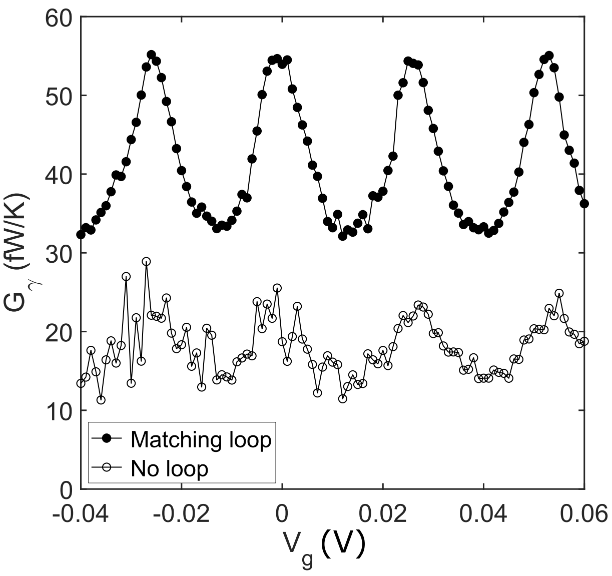

Supplementary Figure 2. **Control experiment and comparison with the "loop" configuration.** Measured source-drain thermal conductance as a function of the gate voltage with (full dots) and without (empty dots) a loop-closing wire, for comparable median temperatures $T_{\rm m} = 187$ mK (loop) and 190 mK (no loop).

lead to a decoupling between electron temperatures in the resistors and the cryostat one below roughly 160 mK. This rather large temperature is well accounted by the small volume Ω of the resistors, for which the external heat load is less efficiently evacuated to the phonon bath.

Control experiment. To confirm further that the heat flow is indeed photonic in nature, we have performed a control experiment where the loop bond closing the circuit is absent, to reduce the current fluctuations responsible for photonic heat flow [8], while still allowing diffusive heat transport with quasiparticles [7]. As shown in Supplementary Figure 2, a clear reduction of the conductance is observed, down to ≈ 45 % of its value and oscillation contrast in the matched situation. The oscillations remain $2e$-periodic: therefore, the remaining heat flow is likely due to capacitive leakage of the connecting apparatus, leading to only partial high-pass filtering of thermal fluctuations and allowing some flow within a residual bandwidth. The observed reduction nonetheless confirms that the source-drain thermal conductance is dominated by the electron-photon coupling, that creates a remote electron-electron coupling with gate-tunable strength.

Estimate for quasiparticle heat transfer. The systematically observed $2e$ periodicity seem to rule out heat transport dominated by a quasiparticle diffusion mechanism, which should be $1e$ periodic. In addition, the measured subgap resistance is $R_{\rm s} \approx 2.5$ MΩ. Applying Wiedemann-Franz law, which should hold at least within a numerical factor of order unity for our CPT [9], we obtain a thermal conductance $G_{\rm qp} \approx \mathcal{L}_0 T/R_{\rm s} \approx 2$ fW/K at 200 mK ($\mathcal{L}_0 = 2.44 \times 10^{-8}$ W.Ω.K^{-2} is the Lorenz number), well below the measured one both in matched and mismatched configuration. Furthermore, the electronic heat conduction is strongly attenuated along the superconducting line due to the reduced quasiparticle density [8], and thus the effective quasiparticle thermal conductance will be much smaller than this upper limit. This rules out for good the hypothesis of quasiparticle-mediated heat transfer. Note that this is different from quasiparticle poisoning, whose effect is to shift the induced charge by an amount e after a quasiparticle tunneling event, which results in averaging the thermal conductance over its value at $n_{\rm g}$ and that at $n_{\rm g} + 1$ (see below).

SUPPLEMENTARY NOTE 1

CPT Hamiltonian and critical current derivation. The Hamiltonian of the Cooper-pair transistor under zero voltage bias, assuming its two JJ are identical and neglecting quasiparticle excitations, writes:

$$\hat{\mathcal{H}} = E_{\rm c}(\hat{n} - n_{\rm g})^2 - 2E_{\rm J}\cos\hat{\phi}\cos\delta/2, \tag{1}$$

where $\hat{n}$ is the number of extra paired electrons in the island, $n_{\rm g} = C_{\rm g}V_{\rm g}/e$ is the gate charge, $\delta = \delta_1 + \delta_2$ is the total phase across the series connection of JJ and $\hat{\phi} = \delta_2 - \delta_1$ is the phase of the island. We derive all properties over the interval $n_{\rm g} \in [-1, 1]$, knowing that the energy bands are periodic in $2e$. We choose for numerical and theoretical purposes to restrict the charge state basis to the subset $\{|n=-2\rangle, |n=0\rangle, |n=2\rangle\}$. The Hamiltonian of

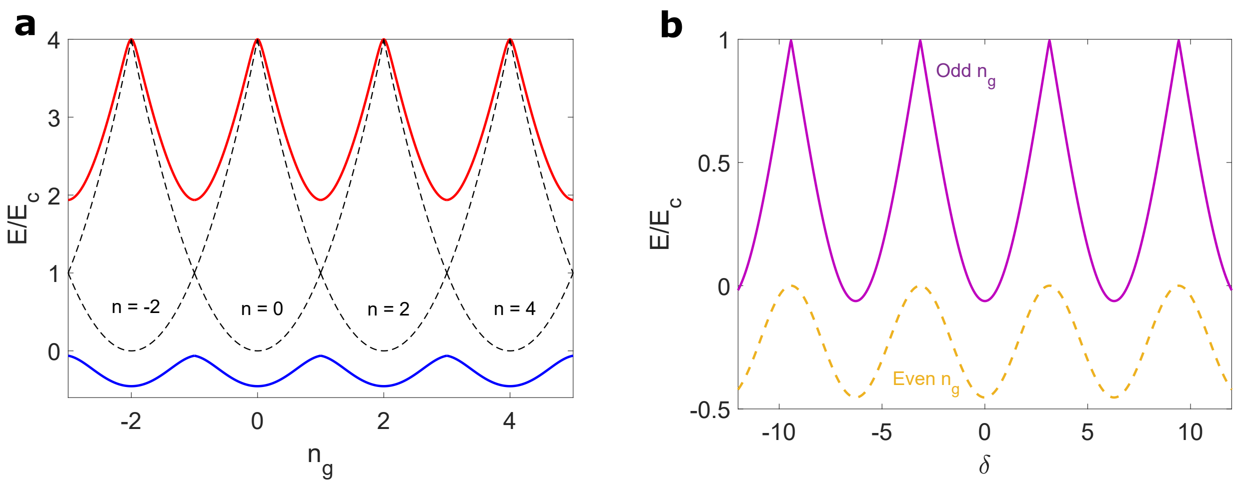

Supplementary Figure 3. **Low energy configuration of the CPT.** a) Ground and first excited energy levels $|E_0\rangle, |E_1\rangle$ of the CPT as a function of the gate charge n_g for $\delta = 0$ (the dotted line corresponds to bare charge states) b) Ground band as a function of the total phase δ accross the CPT for $n_\mathrm{g} = 0$ (yellow) and 1 (purple) mod 2.

Supplementary Equation 1 writes in matrix form in the chosen basis:

$$\hat{\mathcal{H}} = \begin{pmatrix} E_\mathrm{c}(2+n_\mathrm{g})^2 & -E_\mathrm{J}\cos\delta/2 & 0 \\ -E_\mathrm{J}\cos\delta/2 & E_\mathrm{c}n_\mathrm{g}^2 & -E_\mathrm{J}\cos\delta/2 \\ 0 & -E_\mathrm{J}\cos\delta/2 & E_\mathrm{c}(2-n_\mathrm{g})^2 \end{pmatrix}. \tag{2}$$

While the full Hamiltonian possesses analytical eigenenergies based on Mathieu functions [10], those are not easy to handle for our analysis. One can show [11] that the restriction to the three lowest charge states provides fairly convenient, analytical expressions for the three first eigenenergies $E_m, m = 0, 1, 2$ (Bloch bands) that are still good approximations of the full theory for $E_\mathrm{c} \sim E_\mathrm{J}$. After diagonalization of the truncated Hamiltonian presented in Supplementary Equation 2, the eigenenergies are:

$$E_m(n_\mathrm{g}, \delta) = \left(\frac{8}{3} + n_\mathrm{g}^2\right) E_\mathrm{c} + \sqrt{4\lambda(\delta)} \cos\left[\frac{\theta(n_\mathrm{g}, \delta) + 2\pi(m+1)}{3}\right], \tag{3}$$

where:

$$\lambda(\delta) = \frac{2E_\mathrm{J}}{3} \cos^2\left(\frac{\delta}{2}\right)(E_\mathrm{J} + 8E_\mathrm{c}), \tag{4}$$

and

$$\theta(n_\mathrm{g}, \delta) = \arccos\left(-\frac{4E_\mathrm{c}E_\mathrm{J}^2\cos^2(\delta/2) + 64E_\mathrm{c}^3(1/9 - n_\mathrm{g}^2)}{3\lambda(\delta)^{3/2}}\right). \tag{5}$$

A representation of the eigenenergy bands along some chosen axes is shown in Supplementary Figure 3. In particular, the charging energy term changes the shape of the effective Josephson potential represented by the ground band, as well as its barrier height.

From then on, the critical current can be derived from the supercurrent operator $\hat{I} = (2e/\hbar)\partial\hat{\mathcal{H}}/\partial\delta$:

$$I_\mathrm{C}(n_\mathrm{g}) = \frac{2e}{\hbar} \max_\delta \left\langle \frac{\partial\hat{\mathcal{H}}}{\partial\delta} \right\rangle \approx \frac{2e}{\hbar} \max_\delta \frac{\partial E_0(n_\mathrm{g}, \delta)}{\partial\delta}. \tag{6}$$

Here the brackets denote thermal average. For $E_\mathrm{J} \gtrsim k_\mathrm{B}T$, one can ignore the second excited state $|E_2\rangle$, but for $T \approx 100 - 200$ mK, there actually is a substantial probability to thermally populate the first excited state $|E_1\rangle$. However, for $E_\mathrm{c} \sim E_\mathrm{J}$ this is true only around the charge degeneracy points, i.e. for odd values of n_g, where the energy splitting $E_1 - E_0$ is minimum. For odd gate charge values, the maximum critical current in the ground or the excited state is the same for our symmetric model, and differs weakly for even moderate junction asymmetry,

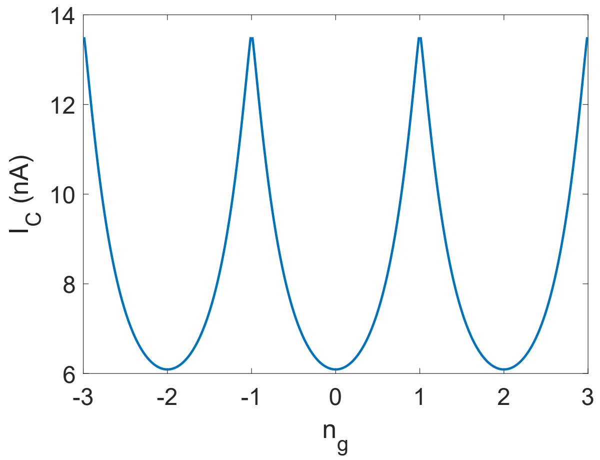

Supplementary Figure 4. **Critical current.** Theoretical critical current as a function of reduced gate voltage, obtained using Supplementary Equation 6.

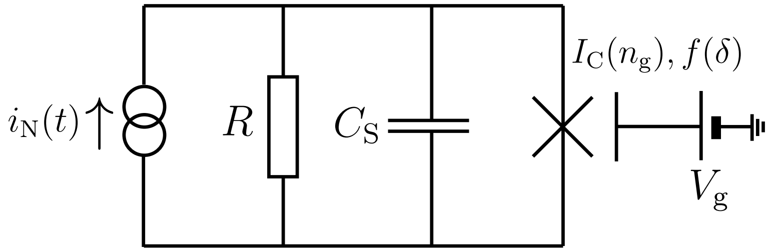

Supplementary Figure 5. **RCSJ model representation.** Equivalent circuit in the loop configuration, with C_{S} the CPT series capacitance, R the series connection of the two resistors generating a Johnson-Nyqvist noise current $i_{\mathrm{N}}(t)$ and the CPT approximated as a single JJ with tunable critical current (/Josephson coupling).

hence the approximation made in the second equality. Using the Ambegaokar-Baratoff relation for E_{J}, the theoretical critical current can thus be readily obtained, as shown in Supplementary Figure 4.

The calculated critical current is significantly higher than the measured switching current, which is affected by thermal escape processes when biased. However, we stress that the value to be used in our modeling is I_{C}, since we are leaving the junction unbiased in the heat transport experiment: what matters here is the plasma frequency, that is, the potential curvature at a minimum, which is defined through I_{C}.

Linear approximation for heat transfer model. In the loop configuration, the total phase δ sits indifferently in one of the local minima of the anharmonic effective Josephson potential, which are all at the same energy since there is no applied voltage or current bias. Due to the environment, δ experiences fluctuations around the local minimum such that its dynamics may be sensitive to the anharmonicity of the potential. A strong anharmonicity can therefore weaken the approximation of the Josephson device as a LC resonator, which is prerequisite to use the filter picture based on an effective inductor used in the Main text and explained in the subsection below. One can give a simple magnitude estimate of the fluctuations, starting from Langevin equation for δ.

In the framework of the RCSJ model [12], we approximate the CPT as a single JJ with a modified current-phase relation (i.e. effective potential) in a RC environment and biased only by the current noise i_{N} due to the series resistor $R = R_1 + R_2$, with spectral density $S_I(\omega) = (2\hbar\omega/R)\coth(\hbar\omega/2k_{\mathrm{B}}T)$ [see Supplementary Figure 5].

Introducing the inverse time constant $\gamma = 1/RC_{\mathrm{S}}$, where $C_{\mathrm{S}} = C/4$ is the series capacitance of the JJ (assuming identical junctions):

$$m\ddot{\delta} + m\gamma\dot{\delta} + f(\delta) = f_{\mathrm{N}}(t), \tag{7}$$

where $m = \hbar^2 C_{\mathrm{S}}/4e^2$, $f(\delta) = \partial E_0/\partial\delta$, and $f_{\mathrm{N}}(t) = \hbar i_{\mathrm{N}}/2e$. The dynamics of δ around zero is that of an anharmonic oscillator with effective mass m. Linearizing around the equilibrium position $\delta = 0$, one obtains the equation of a harmonic oscillator with restoring potential $U(\delta) = m\omega_{\mathrm{p}}^2\delta^2/2$, where $\omega_{\mathrm{p}} = \sqrt{2eI_{\mathrm{C}}/\hbar C_{\mathrm{S}}}$ is the effective JJ plasma frequency. To obtain the typical variance of phase fluctuations, one can make the following observation: in the matched loop configuration, the only capacitance that contributes is that of the series junctions, which is very small ($C_{\mathrm{S}} = 0.36$ fF). Therefore, the oscillator is overdamped ($\omega_{\mathrm{p}}/\gamma \sim 0.1$), and $\omega_{\mathrm{p}} \sim 10^{11}$ rad.s^{-1}. The resulting hierarchy of typical

energies $k_\mathrm{B}T \ll \hbar\omega_\mathrm{p} \ll \hbar\gamma$ allows us to approximate the noise current in the zero temperature limit ($S_I \approx \hbar\omega/R$) while neglecting the acceleration term $m\ddot{\delta}$ in Supplementary Equation 7. As a result:

$$\langle\delta^2\rangle \approx \frac{\hbar^3}{2e^2m^2R\gamma^2}\int_0^{\omega_\mathrm{h}}\frac{\mathrm{d}\omega}{2\pi}\frac{\omega}{\omega^2+\omega_\mathrm{p}^4/\gamma^2} = \frac{R}{R_\mathrm{Q}}\ln\left(1+\frac{\omega_\mathrm{h}^2}{\omega_\mathrm{p}^4/\gamma^2}\right), \tag{8}$$

where $R_\mathrm{Q} = \pi\hbar/2e^2 \approx 6.45$ kΩ is the superconducting resistance quantum and ω_h is a cut-off frequency that is set to $\omega_\mathrm{h} = \gamma$ so as to satisfy the approximation $m\ddot{\delta} \approx 0$. One then obtains a good estimate, for $\gamma/\omega_\mathrm{p} \gg 1$, of the typical spreading of the phase fluctuations:

$$\langle\delta^2\rangle \approx \frac{4R}{R_\mathrm{Q}}\ln\frac{\gamma}{\omega_\mathrm{p}}, \tag{9}$$

which is small for a low impedance environment $R \ll R_\mathrm{Q}$, hence the approximation made in the Main text.

Landauer formula for the heat flow and thermal conductance. The spectrum of the Johnson-Nyqvist voltage noise u_i of the resistor R_i at temperature T_i writes:

$$S_{u_i}(\omega) = 2R_i\hbar\omega\coth\left(\frac{\hbar\omega}{2k_\mathrm{B}T_i}\right), \tag{10}$$

where we have included the quantum statistical cut-off that is a consequence of the Bose distribution of thermal photons. In the classical limit one retrieves the original expression $S_{u_i}(\omega) = 4k_\mathrm{B}T_iR_i$. This voltage noise is modeled as a source in series with the resistor [see Fig. 1c) of the main text]. If the circuit is electrically closed, with a global series impedance $Z_\mathrm{tot}(\omega) = R_1 + R_2 + Z_\mathrm{ext}(\omega)$, the noise current spectrum due to resistance R_i flowing into the loop writes:

$$S_{I_i}(\omega) = \frac{S_{u_i}(\omega)}{|Z_\mathrm{tot}(\omega)|^2}. \tag{11}$$

The Joule power per unit bandwidth dissipated in resistor j due to the noise current emitted by resistor i is $\dot{q}_{i\to j}(\omega) = R_jS_{I_i}(\omega)$. One can write equally the heat dissipated in the reverse direction $\dot{q}_{j\to i} = R_iS_{I_j}(\omega)$. After integrating over all frequencies the resulting net Joule power per unit bandwidth $\dot{q} = \dot{q}_{1\to 2} - \dot{q}_{2\to 1}$, one obtains the net average heat power $\dot{Q}_\gamma$ due to thermal photons exchanged between the two resistors:

$$\dot{Q}_\gamma = \int_0^\infty \frac{\mathrm{d}\omega}{2\pi}\tau(\omega)\hbar\omega\left[n_1(\omega) - n_2(\omega)\right], \tag{12}$$

where $n_i(\omega) = 1/\left[\exp(\hbar\omega/k_\mathrm{B}T_i) - 1\right]$ is the Bose distribution and $\tau(\omega) = 4R_1R_2/|Z_\mathrm{tot}(\omega)|^2$ is the transmission coefficient of the circuit connecting the two resistors. Using the linear approximation as justified above, the total circuit impedance explicitly writes $Z_\mathrm{tot}(\omega, n_\mathrm{g}) = R_1 + R_2 + [\mathrm{i}C_\mathrm{S}\omega + 1/\mathrm{i}L_\mathrm{J}(n_\mathrm{g})\omega]^{-1}$. We can make a further convenient simplification: the plasma frequency for the whole n_g range is about 10^{11} rad.s^{-1}, i.e. $\hbar\omega_\mathrm{p} \gg k_\mathrm{B}T$ throughout all the experiment. As a result, in the angular frequency range of interest (up to $\sim k_\mathrm{B}T/\hbar \sim 2\pi\times 3$ GHz), the capacitive component is irrelevant, and one may retain the Josephson inductance L_J alone to less than a percent accuracy. The series impedance thus simplifies, $Z_\mathrm{tot}(\omega) \approx R_1 + R_2 + \mathrm{i}L_J\omega$, which leads to the definition of a gate-tunable cut-off frequency for thermal radiation imposed by low-pass filtering, $\omega_\mathrm{c}(n_\mathrm{g}) = (R_1 + R_2)/L_\mathrm{J}(n_\mathrm{g})$. On the other hand, for a small temperature difference between R_1 and R_2 and introducing the mean temperature $T_\mathrm{m} = (T_1 + T_2)/2$, one has:

$$n_1(\omega) - n_2(\omega) \approx \frac{\hbar\omega(T_1 - T_2)}{k_\mathrm{B}T_\mathrm{m}^2}e^{\hbar\omega/k_\mathrm{B}T_\mathrm{m}}n_\mathrm{m}^2(\omega), \tag{13}$$

where n_m refers to Bose factor at temperature T_m. Introducing $x = \hbar\omega/k_\mathrm{B}T_\mathrm{m}$, and the reduced circuit cut-off frequency $x_\mathrm{c} = \hbar\omega_\mathrm{c}/k_\mathrm{B}T_\mathrm{m}$, one finally obtains the photon heat conductance $G_\gamma \equiv \dot{Q}_\gamma/(T_1 - T_2)$ presented in the main text:

$$G_\gamma = \frac{2k_\mathrm{B}^2T_\mathrm{m}R_1R_2}{\pi\hbar(R_1+R_2)^2}\int_0^\infty \mathrm{d}x\frac{x^2e^x}{(e^x-1)^2}\frac{1}{1+x^2/x_\mathrm{c}^2}, \tag{14}$$

which depends only on universal constants or independently measured parameters.

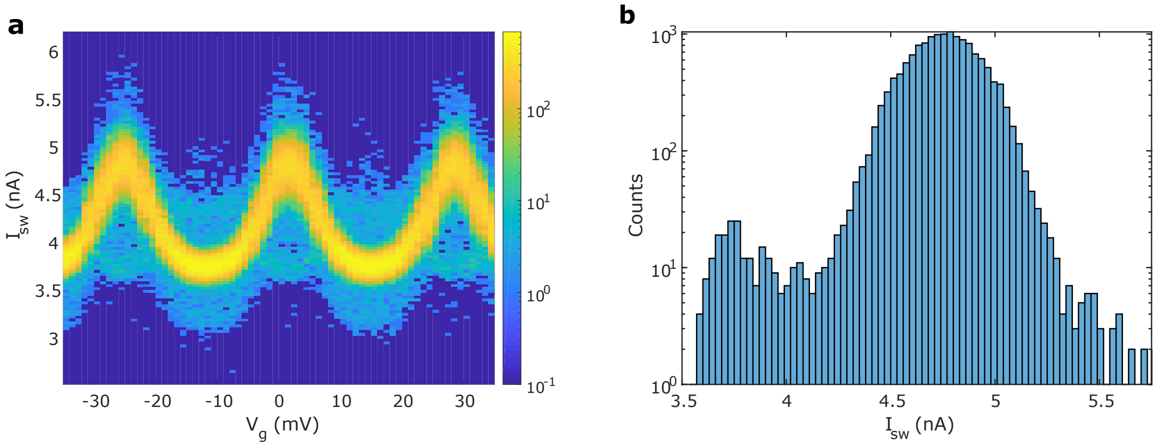

Supplementary Figure 6. **Switching current histograms.** a) Colormap of switching current histograms versus applied gate voltage at 100 mK, for a ramp rate of 10 μA.s^{-1}. The logarithmic scale is intended to reveal poisoning events (the "ghost trace" below each peak), that are barely visible with a linear scale. Each cut is a histogram of switching currents with 5000 attempts. b) Histogram of switching events for $n_g = 1$ mod 2, with data collected from the three cuts at peak maxima.

SUPPLEMENTARY DISCUSSION

In our analysis we have neglected quasiparticle excitations, assuming the island was free of any of it and taking the $2e$ periodicity of our thermal conductance oscillations as an evidence that they do not play a crucial role. Below 200 mK and since $\Delta > E_{\mathrm{c}}$ [13], equilibrium quasiparticles are not expected in the island because of the significant even-odd free energy cost of adding an upaired excitation in the island, $F = -k_{\mathrm{B}}T \ln\left[\tanh\left(D(E_{\mathrm{F}})V_{\mathrm{s}}\sqrt{2\pi k_{\mathrm{B}}T\Delta}e^{-\Delta/k_{\mathrm{B}}T}\right)\right]$ [14] with $D(E_{\mathrm{F}}) = 2.15\times10^{47}$ J^{-1}.m^{-3} the density of states at Fermi level for normal Aluminum and V_{s} the island volume. At a temperature $T^* \approx \Delta/k_{\mathrm{B}} \ln(\Delta D(E_{\mathrm{F}})V_{\mathrm{s}}) \approx 250$ mK, F essentially vanishes and thermal excitations completely spoil the $2e$-periodicity. However, even at our lowest working temperatures (or below in many experiments), an excess, non-equilibrium quasiparticle population possibly due to e.g. stray microwave radiation or non-equilibrium phonons with energies $\gtrsim \Delta$ is commonly observed [15–17], resulting in severe limitations to the coherence of various superconducting devices [16, 18, 19]. We use the three-level model of Supplementary Reference [20], where the states corresponding to zero quasiparticle near or in the island (even parity), to one quasiparticle in the leads near the island (even parity) and to one quasiparticle in the island (odd parity) are, respectively:

$$\begin{aligned} \epsilon_0 &= E_0(n_{\mathrm{g}}, \delta), \\ \epsilon_l &= E_0(n_{\mathrm{g}}, \delta) + \Delta, \\ \epsilon_i &= E_0(n_{\mathrm{g}} + 1, \delta) + \Delta, \end{aligned} \tag{15}$$

We assume that no Cooper pair is broken in the island due to its small volume. In addition, the two Al layers were evaporated with nominally identical settings, so we assume the gaps in the island and in the leads to be equal. Furthermore, we assume a temperature-independent pair breaking rate Γ_{0l} in the regime where non-equilibrium QP dominate (typically well below 250 mK). We call $\delta\epsilon(n_{\mathrm{g}}, \delta) = \epsilon_l - \epsilon_i = E_0(n_{\mathrm{g}}, \delta) - E_0(n_{\mathrm{g}} + 1, \delta)$ the energy difference between the even and the odd parity in presence of a non-equilibrium quasiparticle. In the range n_{g} mod $2 \in [-0.5, 0.5]$, $\delta\epsilon > 0$, hence the island is seen as an energy barrier for QP, that cannot be easily overcome at temperatures $T \ll \delta\epsilon/k_{\mathrm{B}}$. As a result, the probability for a parity switch to occur in this interval is vanishing at low temperatures. On the contrary, in the range n_{g} mod $2 \in [0.5, 1.5]$, $\delta\epsilon < 0$. The island is therefore energetically a "trap" for a quasiparticle excitation: after a pair breaking event creating a quasiparticle near the CPT, a tunneling of the QP in the island will be favourable energetically. As a result, an unpaired excitation may tunnel into the island at a rate Γ_{li} (even to odd parity switch) faster than our measurement times. Such an event leads to a shift by an amount e of the gate charge seen by the noise current flowing through the CPT, after which the quasiparticle may tunnel out by thermal activation at a rate $\Gamma_{il} = \Gamma_{li}e^{\delta\epsilon/k_{\mathrm{B}}T}$. At temperatures $T \ll \delta\epsilon/k_{\mathrm{B}}$, a QP that has entered the island could thus be trapped there for a long time and substantially affect the parity occupation probabilities. To assess qualitatively the magnitude of quasiparticle poisoning in our device, we have measured in the characterization run (no loop) the switching current of the CPT as a function of the gate voltage for several temperatures [20–22].

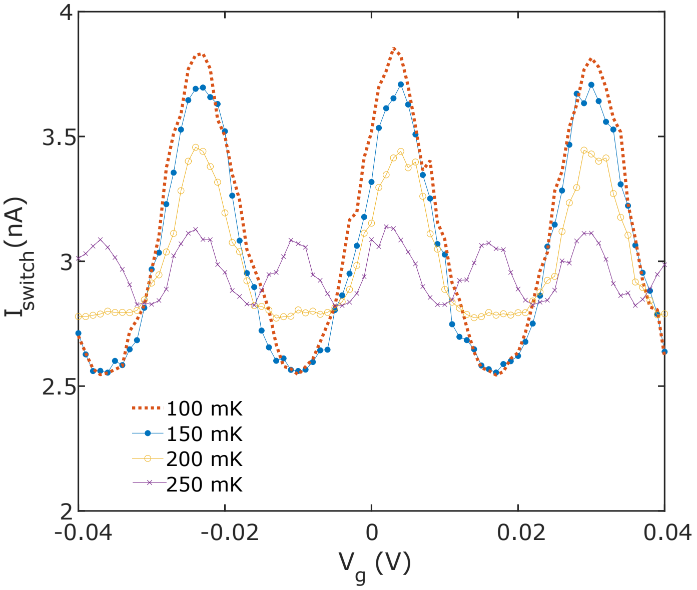

Supplementary Figure 7. **Switching current modulation versus applied gate voltage for different cryostat temperatures.** Each point is the average of 100 switching events.

To obtain a good estimate of the even/odd occupation probabilities, we ramp the current flowing through the CPT beyond the switching current with rates ranging from 100 nA.s^{-1} to 10 μA.s^{-1}. If the ramp is fast enough, then by repeating the procedure $\sim 10^3 - 10^4$ times, we obtain a "snapshot" of the even and odd states occupation, and the switching current distribution typically features a bi-modal distribution. In Supplementary Figure 6b) we have an example of such a distribution, yet it is difficult to obtain with a good accuracy the odd state occupancy due to the significantly low number of corresponding switching events. Besides, the switching current technique suffers from its limited bandwidth set by the finite time of switching to the voltage state of the CPT (here measured to be around 500 μs for all temperatures), which results in several events that lie in between $I_{\mathrm{sw,odd}}$ and $I_{\mathrm{sw,even}}$. In addition, our elevated operation temperatures cause broadening of the histograms [23], which limits further our ability to put a clear separation between odd and even parity events. Therefore, a quantitative comparison with the model developed in Supplementary Reference [20] is not possible for our results. Nevertheless, we can put a lower bound of 96 % on the even number occupation probability below 200mK.

The observed bimodal behaviour of the switching currents distribution at a ramp rate of 10 μA.s^{-1} is washed out for lower ramp rates: in such a situation, a poisoning event might occur in between the switching current expected for the odd parity and the one expected for the even parity. This allows us to extract a characteristic poisoning time Γ_{li}^{-1} in the 100 μs range for odd integer n_{g}, where it is expected to be the shortest due to the "QP trap" configuration of the CPT. Since the energy difference $\delta\epsilon$ between parities is typically $E_{\mathrm{J}}/2 \sim 2 - 3.5 k_{\mathrm{B}}T$ at odd integer n_{g} for our temperatures, such a trap is rather shallow and we can estimate that a QP dwells in the island over a typical time Γ_{il}^{-1} in the ms range before tunneling out.

For temperatures approaching 200 mK and above, the free energy cost of adding one quasiparticle in the island is reduced enough for a significant occupation probability of the odd parity state, and poisoning events occur at timescales faster than the switching time. As a result, the switching currents will be weighted auto-averages of switching currents in even and odd states, which here manifests through additional peaks at even integer n_{g} values. Full $1e$ periodicity of the switching current is recovered at $T = 250$ mK (see Supplementary Figure 7), which corresponds to the temperature T^* where the even-odd free energy cost vanishes.

We now turn to consequences of poisoning on our heat transport measurements. The cut-off frequency $\omega_{\mathrm{c}} = (R_1 + R_2)/L_{\mathrm{J}}$ for blackbody radiation lies in the GHz range for all gate positions, and is therefore several orders of magnitude larger than the typical non-equilibrium poisoning rates Γ_{li}, Γ_{il} based on our estimates. As a result, the low-pass filtering characteristic is unchanged by parity switches for noise current frequencies comparable with or smaller than the typical poisoning rates. At frequencies of the emitted radiation in the decade below ω_{c}, which weight the most in the radiation spectrum, the noise current flows through the CPT much faster than the parity switches. As a result, it should "sample" well both parity states, and the measured thermal conductance should be a weighted average of its value at n_{g} and that at $n_{\mathrm{g}}+1$, with weights corresponding to even and odd state probabilities, respectively. Based on our measurements (96 to 98 % probability of having even parity at $n_{\mathrm{g}} = 1$) and the fact that the

poisoning is more likely for odd integer n_g (therefore making those numbers a low bound for even parity probability regardless of n_g), we conclude that poisoning by nonequilibrium QP plays no significant role in our photonic heat transport measurements. Meanwhile, the onset of equilibrium poisoning around 200 mK may explain in part the reduction in the contrast of thermal conductance modulation when T_m approaches 200 mK.

---

[1] J. P. Pekola, K. P. Hirvi, J. P. Kauppinen, and M. A. Paalanen, "Thermometry by arrays of tunnel junctions," *Phys. Rev. Lett.*, vol. 73, pp. 2903–2906, Nov 1994.

[2] M. M. Leivo, J. P. Pekola, and D. V. Averin, "Efficient peltier refrigeration by a pair of normal metal/insulator/superconductor junctions," *Applied Physics Letters*, vol. 68, no. 14, pp. 1996–1998, 1996.

[3] F. Giazotto, T. T. Heikkilä, A. Luukanen, A. M. Savin, and J. P. Pekola, "Opportunities for mesoscopics in thermometry and refrigeration: Physics and applications," *Rev. Mod. Phys.*, vol. 78, pp. 217–274, Mar 2006.

[4] M. L. Roukes, M. R. Freeman, R. S. Germain, R. C. Richardson, and M. B. Ketchen, "Hot electrons and energy transport in metals at millikelvin temperatures," *Phys. Rev. Lett.*, vol. 55, pp. 422–425, Jul 1985.

[5] K. L. Viisanen and J. P. Pekola, "Anomalous electronic heat capacity of copper nanowires at sub-kelvin temperatures," *Phys. Rev. B*, vol. 97, p. 115422, Mar 2018.

[6] L. B. Wang, O.-P. Saira, D. S. Golubev, and J. P. Pekola, "Crossover between electron-phonon and boundary-resistance limits to thermal relaxation in copper films," *Phys. Rev. Applied*, vol. 12, p. 024051, Aug 2019.

[7] F. Giazotto and M. J. Martínez-Pérez, "The josephson heat interferometer," *Nature*, vol. 492, pp. 401–405 –, Dec 2012.

[8] A. V. Timofeev, M. Helle, M. Meschke, M. Möttönen, and J. P. Pekola, "Electronic refrigeration at the quantum limit," *Phys. Rev. Lett.*, vol. 102, p. 200801, May 2009.

[9] B. Dutta, J. T. Peltonen, D. S. Antonenko, M. Meschke, M. A. Skvortsov, B. Kubala, J. König, C. B. Winkelmann, H. Courtois, and J. P. Pekola, "Thermal conductance of a single-electron transistor," *Phys. Rev. Lett.*, vol. 119, p. 077701, Aug 2017.

[10] A. Cottet, *Implémentation d'un bit quantique dans un circuit supraconducteur / Implementation of a quantum bit in a superconducting circuit.* Theses, Université Pierre et Marie Curie - Paris VI, Sept. 2002.

[11] P. Joyez, *Le transistor à une paire de Cooper : un système quantique macroscopique.* PhD thesis, 1995. Université Paris 6.

[12] W. C. Stewart, "Current voltage characteristics of josephson junctions," *Applied Physics Letters*, vol. 12, no. 8, pp. 277–280, 1968.

[13] K. A. Matveev, M. Gisselfält, L. I. Glazman, M. Jonson, and R. I. Shekhter, "Parity-induced suppression of the coulomb blockade of josephson tunneling," *Phys. Rev. Lett.*, vol. 70, pp. 2940–2943, May 1993.

[14] M. Tinkham, *Introduction to Superconductivity.* Dover Books on Physics Series, Dover Publications, 2004.

[15] A. J. Ferguson, N. A. Court, F. E. Hudson, and R. G. Clark, "Microsecond resolution of quasiparticle tunneling in the single-cooper-pair transistor," *Phys. Rev. Lett.*, vol. 97, p. 106603, Sep 2006.

[16] J. M. Martinis, M. Ansmann, and J. Aumentado, "Energy decay in superconducting josephson-junction qubits from nonequilibrium quasiparticle excitations," *Phys. Rev. Lett.*, vol. 103, p. 097002, Aug 2009.

[17] E. T. Mannila, V. F. Maisi, H. Q. Nguyen, C. M. Marcus, and J. P. Pekola, "Detecting parity effect in a superconducting device in the presence of parity switches," *Phys. Rev. B*, vol. 100, p. 020502, Jul 2019.

[18] G. Catelani, J. Koch, L. Frunzio, R. J. Schoelkopf, M. H. Devoret, and L. I. Glazman, "Quasiparticle relaxation of superconducting qubits in the presence of flux," *Phys. Rev. Lett.*, vol. 106, p. 077002, Feb 2011.

[19] D. Ristè, C. C. Bultink, M. J. Tiggelman, R. N. Schouten, K. W. Lehnert, and L. DiCarlo, "Millisecond charge-parity fluctuations and induced decoherence in a superconducting transmon qubit," *Nature Communications*, vol. 4, p. 1913, May 2013.

[20] J. Aumentado, M. W. Keller, J. M. Martinis, and M. H. Devoret, "Nonequilibrium quasiparticles and $2e$ periodicity in single-cooper-pair transistors," *Phys. Rev. Lett.*, vol. 92, p. 066802, Feb 2004.

[21] P. Joyez, P. Lafarge, A. Filipe, D. Esteve, and M. H. Devoret, "Observation of parity-induced suppression of josephson tunneling in the superconducting single electron transistor," *Phys. Rev. Lett.*, vol. 72, pp. 2458–2461, Apr 1994.

[22] J. Männik and J. E. Lukens, "Effect of measurement on the periodicity of the coulomb staircase of a superconducting box," *Phys. Rev. Lett.*, vol. 92, p. 057004, Feb 2004.

[23] A. Barone and G. Paternò, *Physics and applications of the Josephson effect.* UMI Out-of-Print Books on Demand, Wiley, 1982.
